# Role of p53 in Anticancer Drug Treatment- and Radiation-Induced Injury in Normal Small Intestine

**DOI:** 10.3969/j.issn.2095-3941.2012.01.001

**Published:** 2012-03

**Authors:** Shi Jin

**Affiliations:** Division of Gastroenterology and Hepatology, Department of Medicine, School of Medicine, The Johns Hopkins University, Baltimore, MD 21210, USA

**Keywords:** chemoradiotherapy, genes, p53, DNA damage, small intestine

## Abstract

In the human gastrointestinal tract, the functional mucosa of the small intestine has the highest capacity for absorption of nutrients and rapid proliferation rates, making it vulnerable to chemoradiotherapy. Recent understanding of the protective role of p53-mediated cell cycle arrest in the small intestinal mucosa has led researchers to explore new avenues to mitigate mucosal injury during cancer treatment. A traditional p53 inhibitor and two other molecules that exhibit strong protective effects on normal small intestinal epithelium during anticancer drug treatment and radiation therapy are introduced in this work. The objective of this review was to update current knowledge regarding potential mechanisms and targets that inhibit the side effects induced by chemoradiotherapy.

## Introduction

Although anticancer drug treatment and radiation therapy have been proven to successfully treat many malignancies, including leukemia and lymphoma, or to be complementary remedies for other cancers, their side effects on normal tissue, especially the hematopoietic (HP) system and the epithelium of the gastrointestinal (GI) tract, significantly limit their effectiveness ^[^^1^^]^.

Normal absorption of nutrients is highly dependent on the functional mucosa of the small intestine, which processes one of the highest turnover rates in mammalian tissue ^[^^2^^]^. This property makes small intestinal mucosae vulnerable to chemoradiotherapy. Excessive use of cytotoxic agents and/or radiation has shown that intestinal epithelial cell injury is one of the main factors that lead to GI syndrome, which includes diarrhea, general malabsorption, and infection ^[^^3^^]^. These severe complications not only cause discomfort in patients undergoing chemoradiotherapy but also significantly limit the dose of therapeutic agents. Therefore, preventing cell injury in normal intestinal epithelia is an important therapeutic strategy for cancer treatment.

During chemoradiotherapy, DNA damage in normal cells activates stress-induced signaling pathways, such as p53 ^[^^4^^]^, NF-κB ^[^^5^^]^, reactive oxygen species ^[^^6^^]^, and c-Jun N-terminal kinase (JNK) ^[^^7^^]^. These signaling pathways are amplified, ultimately contributing to normal tissue damage via apoptosis ^[^^8^^,^^9^^]^, necrosis ^[^^10^^]^, autophagy ^[^^11^^]^, or a combination event. Understanding the role of p53 in cell injury or death of normal tissue will provide helpful clues about the mechanisms that could counteract the unavoidable side effects of cancer treatment, and the task remains a challenge for future investigations. Recent advances in cell death have been presented in some excellent review articles ^[^^12^^-^^14^^]^. The present review mainly focused on research advances in the role of p53 in the injury of normal small intestine during anticancer drug treatment and radiation.

## The Small Intestinal Epithelium and Its Stem Cells

### The intestinal epithelium

Efficient digestion of food and absorption of nutrients (carbohydrates, proteins, and lipids) occur primarily in the small intestine. The surface of the small intestine is lined by a single layer of columnar epithelium. The flask-shaped invaginations are called crypts, whereas the finger-like luminal protrusions are termed villi. Six or more independent crypts surround a single villus, resulting in an equal number of parallel columns of epithelial cells running toward the villus tip. Four cell types comprise the small intestinal epithelium, namely, enterocytes, goblet cells, enteroendocrine cells, and Paneth cells ^[^^15^^]^. The main functions of enterocytes are digestion, absorption, and secretion. Differentiated enterocytes, the predominant component of the villi, undergo apoptosis to maintain normal gut epithelial function ^[^^16^^]^. Mucus secreted by goblet cells plays a protective role for the mucosa ^[^^17^^]^. Enteroendocrine cells are specialized endocrine cells scattered throughout the small intestinal mucosa. Four major GI hormones are secreted by enteroendocrine cells: secretin, gastrin, cholecystokinin, and gastric inhibitory peptide ^[^^18^^]^. Paneth cells are known to be important in immunity and host defense for maintaining the mucosal barrier ^[^^19^^]^.

### Stem cells within intestinal crypts

**Figure 1:**
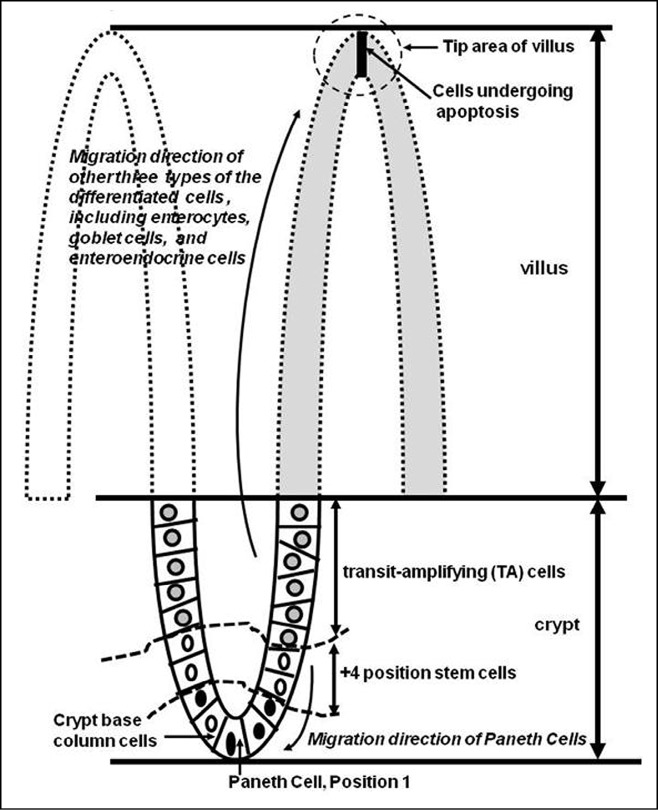
Model for the cell lineage in small intestinal crypts. The small intestinal epithelium consists of two main structures: villi (luminal protrusions) and crypts (invaginations). The villi are composed of fully differentiated cells that mediate absorption and secretion. Apoptosis of the cells at the tip of villi and the proliferation of crypt cells maintain the number of functional cells in the small intestinal epithelium. All cell types within an individual crypt are sustained by stem cells located at the bottom of the crypt. Two stem cell positions have been reported for crypts: One represents +4 position stem cells ^[^20^]^ (shown between the dotted curve lines), and the other refers to CBC cells located between differentiated Paneth cells ^[^21^]^. Once TA cells divided from the stem cells reach the crypt–villus junction, they differentiate and migrate to the villi.

The above-mentioned types of differentiated villus cells are derived from common stem cells within intestinal crypts. Historically, stem cells are hypothesized to be located at approximately cell position 4, named “+4 position stem cells”, from the crypt bottom of the small intestine. The first three positions are occupied by terminally differentiated Paneth cells (**Figure 1**) ^[^^20^^]^. Recent research using a genetic approach has shown that cycling crypt base columnar (CBC) cells are actually located in the “stem cell zone” and interspersed between Paneth cells. CBC cells are positive for Lgr5 (leucine-rich repeat-containing G-protein-coupled receptor 5) expression ^[^^21^^]^.

The small intestinal epithelial layer maintains a steady state by dynamically replenishing lost cells at the tip of villi with proliferating cells derived from stem cells through migration and differentiation (**Figure 1**). When stem cell division occurs, a transit-amplifying (TA) cell is generated and the second daughter cell replaces the parent stem cell. TA cells typically undergo a limited number of cell divisions. When the committed TA cells reach the crypt–villus junction, they differentiate irreversibly^[^^20^^]^. In mice, replacement of the entire population of small intestinal epithelial cells takes 5 days ^[^^22^^]^. The differentiation process of cells operates by bi-directional migration. As shown in **Figure 1**, enterocytes, goblet cells, and enteroendocrine cells, which are within the base of crypts, become differentiated as they move upward to the tip of villi, whereas Paneth cells migrate to the bottom of the crypt. Thus, the whole epithelial sheet is in continuous movement.

Apoptosis for genetic integrity in the stem cell compartment is one of the key mechanisms accounting for the rare development of cancer in the small intestine. Potten ^[^^23^^]^ reported that the +4 position cells are extremely sensitive to radiation, which functionally protects the stem cell compartment from genetic damage. In this proposed model, stem cells with DNA damage are replaced by the first two to three generations of TA cells. With better repair capacity, these TA cells fall back into the +4 position and, more importantly, may potentially regain the properties of their parent stem cells. Evidence demonstrating that crypt cell death precedes villous atrophy and loss of differentiated enterocytes during cancer therapy has been reported, indicating that inappropriate loss of crypt cells, especially stem cells, is the primary cause of GI tract damage ^[^^24^^]^.

## The p53 Protein and Its Downstream Pathways

**Figure 2:**
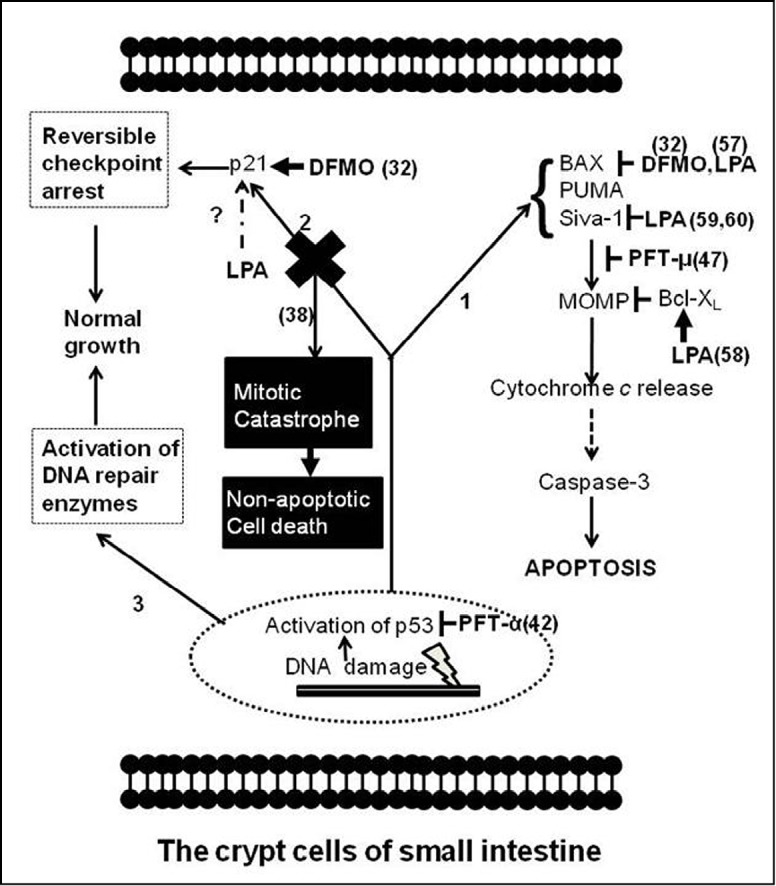
Schematic representation of p53 signaling in response to anticancer drug treatment and radiation therapy in crypt cells of the small intestine and action sites of 3 molecules (PFT, DFMO, and LPA). In response to DNA damage, p53 accumulation leads to the activation of three pathways involved in (1) apoptosis, (2) cell cycle arrest, and (3) DNA repair. The transcriptional activity of p53 increases the expression levels of proteins responsible for the three pathways, such as p21 and Bax. The cytosol function of p53 also directly induces the MOMP. PFT-α inhibits the transcriptional activity of p53 ^[^28^]^, whereas PFT-µ binds p53 to attenuate the binding affinity of anti-apoptotic proteins, such as Bcl-X_L_^[^29^]^. DFMO increases the expression of p21 but inhibits that of Bax ^[^30^]^. LPA blocks the translocation of Bax from cytosol to mitochondria ^[^31^]^, accelerates the protein degradation of pro-apoptotic Siva-1 ^[^32,33^]^, and increases the protein expression of Bcl-X_L_^[^34^]^. The effect of LPA on p21 in response to DNA damage is currently unknown. In some conditions, such as high-dose radiation for cancer treatment, blocking p53 (such as in p53 KO mice) in crypts of the small intestine leads to mitotic catastrophe, a type of cell death occurring during mitosis, as a result of DNA damage ^[^35^]^. Inhibiting the p53-mediated p21 pathway is a major mechanism responsible for mitotic catastrophe in cells with unrepaired DNA. The p53-mediated cell cycle arrest pathway is hypothesized to offer cells a time window to process DNA repair in response to DNA damage. The numbers within parentheses indicate the sources of these findings. Detailed mechanisms related to apoptosis have been reviewed elsewhere ^[^12-14^]^.

The p53 gene encodes protein containing 393 amino acid residues ^[^^25^^]^. In non-stressed cells, p53 has a short half-life (~20 min), and its cellular concentration is thus maintained at a relatively low level ^[^^26^^]^. When the cells are stressed by events from inside or (and) outside the cells, such as DNA damage induced by anticancer drug treatment or radiation therapy, p53 dissociates from its binding partner, MDM2, a critical negative regulator of p53, and is activated by post-translation modification ^[^^27^^]^. As shown in **Figure 2**, p53 activation leads to G1-phase cell cycle arrest and DNA repair via transcriptional upregulation of related genes, such as p21 ^[^^36^^]^. Successful DNA repair will allow cells to proceed with the cell cycle. Under DNA damage conditions, p53 also increases the expression of genes responsible for cell apoptosis, such as Bax ^[^^37^^]^ and p53 upregulated modulator of apoptosis (Puma) ^[^^38^^]^.

In short, the core function of p53 in normal cells is as a guardian to monitor and maintain the integrity of the genome. Most human cancers have been observed to exhibit defects in p53, indicating the key role of p53 in tumor suppression ^[^^27^^]^. On one hand, restoration of wild-type (WT) p53 in p53-null tumors can be speculated to turn on the p53-mediated pathways to eliminate or at least inhibit the growth of tumors during cancer treatment. On the other hand, during chemoradiotherapy for p53-deficient tumors, temporarily blocking p53 function might significantly reduce injury in normal tissues bearing intact WT p53 without the potential problem of increasing the risk of tumorigenesis.

## Role of p53 in Anticancer Drug Treatment-Induced Small Intestinal Epithelial Cell Injury

The role of p53 in response to anticancer drug treatment has been recently extensively investigated in normal small intestinal epithelial cells. The findings from such studies provide important clues for finding new molecules to counteract chemoradiotherapy-induced side effects on the intestinal epithelium. Some studies used the IEC-6 cell line, which is derived from rat normal small intestine, as a working *in vitro* model. According to morphological and immunological criteria, IEC-6 cells are specifically derived from intestinal crypt cells with intact p53. They are nontumorigenic and retain the undifferentiated character of epithelial stem cells ^[^^39^^]^. Camptothecin (CPT; topoisomerase-1 inhibitor; 20 µM) has been shown to induce DNA double-strand breaks and activate the ataxia telangiectasia mutated kinase (ATM kinase)/ataxia telangiectasia and Rad3-related kinase (ATR kinase)/p53 signaling axis. Activated ATM/ATR phosphorylates p53, which helps inhibit p53 degradation. Accumulation of p53 in cells accelerates the synthesis of pro-apoptotic Bax and lowers the protein level of anti-apoptotic Bcl-X_L_. The increased ratio of pro-apoptotic Bcl-2 family proteins to anti-apoptotic Bcl-2 family proteins leads to mitochondrial outer membrane permeabilization (MOMP). Subsequently, mitochondrial release of cytochrome c activates caspase-9 and casaspe-3, eventually resulting in cell death ^[^^30^^]^.

Several interesting points need to be noted and discussed. First, previous studies have shown that inhibition of the ATM/ATR kinases by their inhibitor (CGK733) completely prevents CPT-induced apoptosis, suggesting that upstream kinases of p53 might be the therapeutic targets for interference of p53-induced cell death pathways. Second, as the ratio of pro-apoptotic Bcl-2 family proteins to anti-apoptotic Bcl-2 family proteins has been observed to be altered in p53-induced cell death, mechanisms to reverse the ratio of these proteins will provide alternative strategies against cell death triggered by p53. Third, protein synthesis inhibition by cycloheximide is known to be required for tumor necrosis factor (TNF) a-induced apoptosis in IEC-6 cells, whereas a recent study demonstrated that the combination modality of TNF-a and CPT leads to robust activation of caspase-8 as well as JNK and cell death ^[^^40^^]^. JNKs are the key pro-apoptotic kinases of the small intestinal epithelium, especially in death receptor-induced apoptosis ^[^^41^^,^^42^^]^. The immune system of patients undergoing chemotherapy is frequently compromised. Under this condition, activated monocytes and macrophages can be speculated to increase the release of pro-inflammatory mediators, such as TNF-a. Moreover, CPT analogues have been shown to directly induce TNF-a production in monocytes ^[^^43^^]^. Although the precise mechanism by which p53 activates caspase-8 and JNK remains unknown, the above-cited data suggest that blocking of p53 or one of its downstream pathways might reduce death receptor- and DNA damage-induced intestinal cell injury during anticancer drug treatment.

## The Complicated Role of p53 in Radiation-Induced Small Intestinal Cell Injury

The current understanding of the role of p53 in radiation-induced small intestinal injury is based on studies of mice exposed to whole-body radiation (WBR). Overall, the strength of radiation determines the fate of small intestinal epithelial cells (especially the potential stem cells), for example, cell cycle arrest, senescence, or apoptosis.

In the absence of radiation, natural spontaneous apoptosis occurs in potential stem cells. This type of p53-independent apoptosis is a mechanism for guarding genomic integrity regarded as one way of inhibiting tumorigenesis. Low-dose radiation (<1 Gy gamma irradiation) results in peak levels of apoptosis 3-6 h post-radiation. Merritt et al. ^[^^44^^]^ reported that the p53 knockout (KO) mice exposed to 8 Gy of radiation in their study did not have detectable apoptosis in the base of crypts, suggesting an apoptotic role for p53.

p53 likely plays a similar role with increased radiation. However, the data accumulated from p53 KO mice suggest that p53 plays a survival role in small intestinal epithelial cells at higher levels of radiation. Komarova et al. ^[^^35^^]^ reported noteworthy observations. First, p53 KO mice exposed to less than 10 Gy of radiation had a higher survival rate compared with WT mice. Unexpectedly, p53 KO mice treated with higher doses of radiation (>12.5 Gy) were more sensitive to radiation and died much sooner compared with WT mice. Second, when mice were treated with 15 Gy of radiation, there were no difference in mouse survival rates between WT mice transplanted with WT bone marrow and with p53 KO bone marrow, indicating that the HP system has no effect on the increased sensitivity of the intestine of p53 KO mice. Third, decreased amounts of crypts were observed in mice of both genotypes in the first 3 days after radiation treatment. However, most crypts disappeared in p53 KO mice and regenerating crypts became detectable in WT mice on Day 4.

The less extent of small intestinal damage observed in WT mice treated with high-dose radiation compared with p53 KO mice is attributed to the decreased lethal mitotic catastrophe of crypt cells in WT mice via p53-mediated cell cycle checkpoints and DNA repair activation. These findings suggest that p53 has a dual role for small intestinal damage during severe radiation treatment: On one hand, it plays a destructive role via the induction of pro-apoptotic proteins, such as Bax, Bak, and Puma, to promote mitochondria-dependent apoptosis in the early phase (Day 1 after WBR). On the other hand, it plays a protective role by inhibiting lethal mitotic catastrophe via the induction of proteins for cell cycle checkpoints and DNA repair machinery, such as p21, in the late phase (Days 4 and 5 after WBR).

The above-mentioned findings have in fact been confirmed, at least partially, by recent series of work. PUMA KO mice exposed to 15 Gy of radiation exhibited higher levels of p21, enhanced crypt proliferation and regeneration, and prolonged survival rates compared with WT mice ^[^^45^^]^. Interestingly, after WBR, PUMA/p21 double-KO mice demonstrated characteristics of apoptosis inhibition in the early phase and exacerbated GI damage in the late phase ^[^^46^^]^. Furthermore, the protective effect against GI syndrome observed in transgenic mice with an additional copy of p53, named “Super p53 mice”, was reversed by crossing these mice with p21 KO mice, which means that these p21 KO/Super p53 mice were highly sensitized to the GI syndrome compared with the Super p53 mice ^[^^47^^]^. Taken together, these studies suggest that blocking Puma-dependent apoptosis helps preserve the stem and progenitor cell compartments immediately after radiation, whereas maintaining the functional p53-mediated p21 pathway in the late phase enhances DNA repair as well as genome stability and further aids the regeneration and survival of crypt cells.

## Molecules for Alleviating Chemo-radiotherapy-induced Damage to Normal Tissues Including Small Intestine

Many molecules have been extensively studied for developing relatively new ways to inhibit normal intestinal epithelial cell injury during cancer treatment. Based on recent studies on the paradoxical role of p53 in intestinal epithelial cell damage, although p53 is still considered to play a central role in mediating anticancer drug treatment- and radiation-induced damage, the idea that simply blocking p53 protects against normal tissue damage, especially for the small intestine, needs to be revised. Selectively inhibiting p53-dependent cell death response without altering its function for promoting cell cycle arrest may likely effectively reduce damage in the normal intestine. Three molecules for inhibiting normal tissue damage mediated by p53 or any of its downstream pathways during cancer treatment are herein introduced: pifithrin (PFT), a-difluoromethylornithine (DFMO), and lysophosphatidic acid (LPA).

### PFT: a specific inhibitor of p53 protein

PFT-α was first isolated in 1999, and initial studies showed that it successfully protects different tissues and cell types, including endothelial cells ^[^^48^^]^, cardiomyocytes ^[^^49^^]^, and kidney proximal tubular cells ^[^^50^^]^, from damage induced by anticancer drug treatment by inhibiting the transcriptional activity of p53 (**Figure 2**) ^[^^28^^]^. Moreover, PFT-α also has been proven to be an effective agent for radioprotection from HP syndrome ^[^^28^^]^. However, evidence showing that PFT-α does not protect mice from GI syndrome by inhibiting the p53-mediated p21 pathway has also been reported. Komarova et al. ^[^^35^^]^ showed that p21 KO mice treated with high doses of radiation (>12.5 Gy) were more sensitive to high radiation and died from GI syndrome significantly earlier than WT mice, indicating that cellular growth arrest mediated by the p21 pathway, which is regulated by the transcriptional activity of p53, plays a protective role against damage induced by high doses of radiation in the small intestinal epithelium. Encouraged by these results related to the p53-mediated p21 pathway and new evidence showing that the p53 protein directly induces MOMP by binding with the protective Bcl-X_L_ and Bcl-2 proteins ^[^^51^^]^, Strom et al. ^[^^29^^]^ isolated a novel PFT (PFT-m) in 2006. This new-generation PFT selectively inhibits p53-dependent apoptosis by reducing p53 affinity to anti-apoptotic Bcl-X_L_ and Bcl-2 and has no effect on p53-dependent transcriptional activity (**Figure 2**). Another issue that needs to be addressed is the effect of this new compound on the integrity of small intestinal mucosa during anticancer drug treatment and high-dose radiation therapy. The development of both PFT-α (the general inhibitor of p53) and PFT-µ (the inhibitor specifically targeting the pro-apoptotic branch of p53 function) into drugs is an essential research undertaking.

### DFMO: a well-established inhibitor of ornithine decarboxylase

Pharmacologically, DFMO is a well-established irreversible inhibitor of ornithine decarboxylase, which is the first rate-limiting enzyme for the synthesis of intracellular polyamines, namely, putrescine, spermidine, and spermine. The concentrations of intracellular polyamine pools are highly regulated to meet the requirements of normal cell growth and differentiation. The direct effect of DFMO is to deplete the intracellular polyamine pools ^[^^52^^]^.

Polyamine depletion by DFMO protects IEC-6 cells from CPT-induced apoptosis by blocking cytochrome c release from mitochondria and attenuates gamma radiation-induced apoptosis by inhibiting activation of caspase-3, one of the major executive caspases ^[^^53^^,^^54^^]^. In addition, significantly increased intestinal crypt regeneration and higher levels of crypt survival rates were observed in mice orally treated with DFMO compared with control mice under the mouse WBR model ^[^^54^^]^. Recently, Bhattacharya et al. ^[^^30^^]^ dissected the molecular mechanisms by which DFMO protects normal small intestinal epithelial cells in response to cancer treatment. They reported that DFMO increased p21 expression but inhibited apoptotic Bax protein expression, which were not expected (**Figure 2**) ^[^^30^^]^. The expression of both p21 and Bax proteins is controlled by activated p53 at the transcriptional level. Defining the precise mechanisms by which DFMO selectively favors one downstream arm of p53 and simultaneously inhibits another is a very interesting challenge. Although it is currently not easy to pinpoint the exact mechanisms accounting for the bi-directional effects of DFMO on p53-mediated pathways, it seems that some factors prevent the p53-induced synthesis of Bax but others enhance the p53-mediated p21 pathway, which induces cell growth arrest, during polyamine depletion. These observations prompt further questions: What are these factors, and how do they work? Therefore, identifying these factors is critical for dissecting the role of polyamine in p53 pathway modulation.

Recent studies have also shown that polyamine depletion by DFMO significantly inhibits cancer growth^[^^54^^]^. Thus, thoroughly investigating the molecular mechanisms of the double effects of DFMO on normal and tumor tissues and testing the degree of protection that DFMO has on normal small intestine during cancer treatment using a tumor-bearing mouse model could have significant clinical benefits.

### LPA: a phospholipid growth factor

In earlier studies, such polypeptide growth factors as keratinocyte growth factor ^[^^55^^]^, insulin-like growth factor 1 ^[^^56^^]^, interleukin 11 ^[^^57^^]^, and fibroblast growth factor-2^[^^58^^]^ have been shown to protect intestinal stem cells and increase animal survival rates following WBR. In recent years, accumulating evidence has suggested that LPA, a phospholipid growth factor, which is a normal component of blood plasma, has strong potential ability to act against and mitigate the intestinal damage induced by anticancer drug treatment or radiation therapy. The major physiological function of LPA lies in its profound ability to activate specific G protein-coupled family receptors ^[^^59^^]^. Increasing Bax expression and enhancing its activation are key proapoptotic functions of p53. With the IEC-6 cell line as an in vitro model, which mimics intestinal crypt cells, Deng et al. ^[^^31^^,^^60^^]^ reported that LPA protected cells from CPT-induced DNA damage by inhibiting the translocation of Bax from cytosol to mitochondria, suggesting the counteraction of LPA to the p53-mediated pro-apoptotic pathway (**Figure 2**). More importantly, LPA has strong protective effects on mice treated with WBR. When LPA was orally applied to mice 2 h before WBR, the apoptotic body observed in stem cell positions significantly decreased compared with that observed in WT mice ^[^^60^^]^.

In 2007, to improve the effects of LPA for GI protection, using computational and pharmacological approaches, one research group successfully designed a metabolically stabilized LPA mimic, named octadecenyl thiophosphate (OTP). Compared with LPA, OTP is neither cleaved by pancreatic lipase, the major lipase in the intestine, nor inactivated by lipid phosphatase cleavage, another mechanism for the inactivation of LPA, in addition to lipase-induced inactivation. This strong resistance to pancreatic lipase and lipid phosphatase offers OTP a greater opportunity to bind with LPA receptors expressed on the cellular plasma membrane of the intestinal epithelium. Oral administration of OTP 2 h before WBR at a strength of 15 Gy significantly protected intestinal epithelial cells from apoptosis and enhanced survival crypts 4 days after radiation compared with LPA treatment ^[^^34^^]^. Although the precise and detailed mechanism by which OTP modulates p53-related pathways remains unknown, based on a recent study showing that LPA stimulation increases the expression of Bcl-X_L_ (anti-apoptotic protein) and promotes degradation of pro-apoptotic protein Siva-1 (**Figure 2**) ^[^^32^^-^^34^^]^, OTP likely protects the small intestine with altered ratios of anti-apoptotic Bcl-2 family proteins to p53-mediated pro-apoptotic Bcl-2 family proteins. This compound is bound to eventually become a suitable agent for mitigating p53-controlled DNA damage to the small intestine, especially for radiation-induced damage.

## Summary and Future Directions

During cancer treatment, rapid DNA damage induces injury in crypt cells of the small intestine, especially stem cell compartments, followed by damage of villi in the late phase. The possible mechanisms behind this type of damage are apoptotic cell death and non-apoptotic cell death, such as mitotic catastrophe, a type of cell death that occurs during mitosis. Puma has been shown to sufficiently activate Bax by itself, eventually resulting in apoptosis even in the absence of p53, indicating the involvement of a p53-independent mechanism in radiation-induced apoptosis ^[^^61^^]^. Recent understanding of the protective role of p53-mediated cell cycle arrest for the intestinal mucosa opens another window for mitigating mucosal injury during cancer treatment. Simply blocking p53 might protect cells from p53-dependent apoptosis in the early phase and unavoidably leads to cells with high potential for mitotic catastrophe because of their ability to escape cell cycle arrest in the late phase. In this light, future basic biomedical research should focus on elucidating the more deliberated molecular mechanisms by which p53 orchestrates apoptosis, cell cycle arrest, and mitotic catastrophe in normal intestine during cancer treatment. This type of research is expected to lead to the generation of new inhibitors, such as the Puma inhibitor, to block unnecessary cell death without interfering with the p53 function of maintaining genomic stability.
